# Oral squamous cell carcinoma vs. medication-related osteonecrosis of the jaw in patients assuming bone-modifying agents: a diagnostic challenge in a multi-hospital case series

**DOI:** 10.3389/froh.2025.1574425

**Published:** 2025-06-12

**Authors:** Rodolfo Mauceri, Martina Coppini, Gaetano La Mantia, Giuseppe Seminara, Mario Pérez-Sayáns, Corrado Toro, Rita Vitagliano, Hitoshi Kawamata, Toshiki Hyodo, Giuseppe Colella, Vito Rodolico, Giuseppina Campisi

**Affiliations:** ^1^ Department of Precision Medicine in Medical, Surgical and Critical Care, University of Palermo, Palermo, Italy; ^2^ Unit of Oral Medicine and Dentistry for Frail Patients, Department of Rehabilitation, Fragility, and Continuity of Care, Regional Center for Research and Care of MRONJ, University Hospital Palermo, Palermo, Italy; ^3^ Department of Biomedical and Dental Sciences and Morphofunctional Imaging, University of Messina, Messina, Italy; ^4^ Oral Medicine, Oral Surgery and Implantology Unit (MedOralRes Group), Faculty of Medicine and Dentistry, Universidade de Santiago de Compostela, A Coruña, Spain; ^5^ ORALRES group Santiago de Compostela, Instituto de Investigación Sanitaria de Santiago (IDIS), Santiago de Compostela, Spain; ^6^ Instituto de Materiales (iMATUS) de la Universidade de Santiago de Compostela, Santiago de Compostela, Spain; ^7^ Maxillofacial Surgery Unit, Clinica del Mediterraneo of Ragusa, Ragusa, Italy; ^8^ Multidisciplinary Department of Medical-Surgical and Dental Specialties, Oral and Maxillofacial Surgery Unit, University of Campania “Luigi Vanvitelli”, Naples, Italy; ^9^ Department of Oral and Maxillofacial Surgery, Dokkyo Medical University School of Medicine, Sōka, Japan; ^10^ Department of Pathologic Anatomy and Histology, University of Palermo, Palermo, Italy; ^11^ Department of Biomedicine, Neuroscience and Advanced Diagnostics (Bi.N.D), University of Palermo, Palermo, Italy

**Keywords:** oral squamous cell carcinoma (OSCC), squamous cell carcinoma of head and neck, oral cancer, medication-related osteonecrosis of the jaw (MRONJ), osteonecrosis of the jaw (ONJ)

## Abstract

**Background:**

Despite advances in the diagnosis of oral squamous cell carcinoma (OSCC), most cases are diagnosed in advanced stages, influencing negatively the patient's prognosis. The absence of specific signs and symptoms contributes to the diagnostic delay of OSCC, often leading to confusion with various oral conditions, including, in patients with a history of bone modifying agents (BMA) and/or anti-angiogenic (AA) molecules, the Medication-Related Osteonecrosis of the Jaw (MRONJ). This study aims to investigate the characteristics of OSCC and MRONJ, focusing on clinical and radiological features of a multicenter series.

**Methods:**

According to STROBE statements, 11 patients collected by different centers and affected by OSCC undergoing BMA or AA therapy, with clinical and radiological features resembling MRONJ were reported (6 in Italy and 5 in Japan). Due to the suspicion of a malignant neoplasia, incisional biopsies for histological examination were performed.

**Results:**

In all eleven patients under ONJ-associated therapy, discerning between OSCC and MRONJ was a real challenge due to overlapping clinical and radiological features. The present case series highlights the importance of considering the possibility of malignant disease in patients undergoing ONJ-associated therapy.

**Conclusion:**

Although biopsy is commonly considered unnecessary for MRONJ diagnosis, our findings highlight the importance of selectively performing bioptic procedures in patients taking ONJ-associated therapy to exclude the malignant nature of oral lesions promptly.

## 1 Introduction

In recent years, an increase in the incidence of oral cancer has been reported, with approximately 377,713 new cases diagnosed worldwide in 2020 (1). Oral squamous cell carcinoma (OSCC) is the most common type (2, 3). The etiopathogenesis of OSCC is multifactorial, and it has been related mainly to chemical factors (e.g., tobacco, and alcohol) and other factors, such as infections (e.g., human papillomavirus) and genetic alterations (4).

The natural history of OSCC is not fully understood; not all OSCC derived from prior oral potentially malignant disease (OPMD); some OSCC can develop from lesions in which epithelial dysplasia was not previously diagnosed, or from apparently normal mucosa that may contain significant molecular aberrations that increase the likelihood of cancer (5, 6).

Regarding clinical presentation, OSCC has multiple features, sometimes representing a challenge in its identification, especially in the early stages. In the advanced stage, the classic features of OSCC include ulceration, nodularity, and fixation to underlying tissues (7).

In the presence of a questionable lesion of the oral cavity which, after eliminating the possible causal factor, does not improve within 2 weeks or more, the gold standard is biopsy and histopathological examination (8).

The clinical stage at the time of diagnosis is recognized as an important prognostic marker; the differences in 5-year mortality rates based on staging are marked, with >80% survival in those with localized disease, compared with <30% in those with advanced disease (9). The causes of the high mortality rate are attributable to the diagnostic delay which worsens the patient's prognosis. Unfortunately, there are many studies in the literature concerning the diagnostic delay of oral cancer (9, 10). Diagnostic delay may be attributable to patient-related factors (e.g., no visits to the dentist or doctor) and physician-related factors (e.g., lack of a meticulous clinical examination of the oral mucosa) (5, 10). Moreover, of the known factors related to diagnostic delay, the literature suggests that one of the main causes of delay attributed to the patient is a lack of knowledge regarding OSCC and related risk factors. In addition, the absence of the pathognomonic signs or symptoms of OSCC often leads patients to incorrectly attribute these signs or symptoms to infections or dental problems (11). As regards the factors contributing to professional delay, a decisive role can be played by the lack of information, awareness, and specific training of oral health specialists (12).

The diagnostic delay may also be associated with an incorrect differential diagnosis; some authors have hypothesized that in some suspected cases of MRONJ, OSCC diagnosis should be considered and ruled out (13).

MRONJ has been defined as “an adverse drug reaction described as the progressive destruction and death of bone that affects the mandible and maxilla of patients exposed to the treatment with medications known to increase the risk of disease, in the absence of a previous radiation treatment” (13).

The medications known to increase the risk of MRONJ are principally the bone-modifying agents (BMAs), such as bisphosphonates (BPs) and denosumab (DNB), but also, more rarely, some biological medications that have no antiresorptive activity on bone tissue (e.g., tyrosine kinase inhibitors), often assumed concurrently with BMAs (14, 15).

The peculiarity of MRONJ is that, like early OSCC, it is characterized by non-specific clinical and radiological signs, which in some cases can be misleading, delaying its diagnosis (11, 16, 17).

In patients with a prior history of BMA or AA therapy, the onset of a mucosal lesion with or without bone exposure could pose the suspicion of MRONJ, but it is also important to consider the doubt criteria (13). The present study aims to investigate different pictures of OSCC mimicking MRONJ in patients undergoing BMA or AA therapy, with a focus on identifying clinical and radiological features, necessary for the correct definitive diagnosis. We reported eleven cases of OSCC in patients at risk of MRONJ with clinical and radiological features resembling MRONJ.

## 2 Materials and methods

The study was approved by the Institutional Local Ethics Committee of the University Hospital “P. Giaccone” of Palermo, Palermo, Italy (approval number #1/2022). The study was conducted according to the Principles of the Declaration of Helsinki on experimentation involving human subjects and written informed consent was obtained from all participants. The study was performed following the STROBE Statement for Observational Cohort Studies (16). The clinical procedures were performed by four expert clinicians: GC (Giuseppina Campisi), at the Unit of Oral Medicine “V. Margiotta” of the University Hospital “Paolo Giaccone” in Palermo (Italy), GC (Giuseppe Colella), at the Unit of Oral and Maxillofacial Surgery of the University of Campania “Luigi Vanvitelli” in Naples (Italy), CT (Corrado Toro) at the “Clinica del Mediterraneo” in Ragusa (Italy), and HK (Hitoshi Kawamata) at the Dokkyo Medical University School of Medicine of Shimotsuga, Tochigi (Japan) respectively. During the patient’s interview, variables including sociodemographic data, medical history, and previous therapy with MRONJ-related drugs were recorded. All included patients underwent oral examination, dedicated radiological investigation (e.g., orthopantomography—OPT), and, when necessary, computed tomography (CT), cone beam CT (CBCT) or Magnetic Resonance Imaging (MRI), and incisional mucosal biopsy to obtain tissue specimen for the histological diagnosis.

## 3 Results

In the participating centers, 711 cases of suspected MRONJ were observed over the past 5 years, of which 11 were ultimately diagnosed as OSCC after further diagnostic investigation. These eleven patients, all undergoing treatment with bone-modifying agents (BMAs) or antiangiogenic agents (AAs), were consecutively included in the study. The baseline features of the cases are reported in Table 1. There were 10 females (10/11, 90.9%). The mean age was 73 ± 7.9 years (median 72 years). Only one patient was a former smoker (1/11, 9.1%). Eight patients were affected by osteoporosis (8/11, 72.7%), two patients were affected by breast cancer and bone metastases (2/11, 18.2%) and one patient was affected by chronic lymphocytic leukemia (1/11, 9.1%).

**Table 1:** Characteristics of included patients.

Patient	Age	Sex	Systemic disease	ONJ-related drugs	Duration (months)	Cumulative dose (mg)	OSCC localization	Risk factors	Bone exposure
#1	90	F	Osteoporosis	Ibandronate	120	18,000	Mandible	-	No
#2	72	F	Osteoporosis	Alendronate	120	33,600	Mandible	-	No
#3	71	F	Breast cancer and bone metastases	Denosumab^a^	12	1,440	Maxilla	Former smoker	Yes
#4	75	M	Chronic lymphocytic leukemia	Imbrutinib	14	1,76,400	Mandible	-	No
#5	62	F	Osteoporosis	Alendronate	24	6,720	Mandible	-	Yes
#6	64	F	Breast cancer and bone metastases	Denosumab^a^	12	1,440	Maxilla	-	Yes
#7	76	F	Osteoporosis	Risedronate	60	4,200	Mandible	-	No
#8	64	F	Osteoporosis	Minodronate	24	4,800	Mandible	-	No
#9	70	F	Osteoporosis	Minodronate	72	14,400	Mandible	-	No
#10	78	F	Osteoporosis	Minodronate	84	16,800	Mandible	-	No
#11	81	F	Osteoporosis	Alendronate	6	840	Mandible	-	No

^a^ High-doses of denosumab (120 mg every 4 weeks).

Regarding ONJ-related drugs type, two patients received high-doses of denosumab (120 mg sc every 4 weeks) (2/11, 18.1%), three patients used alendronate (70 mg os every week) (3/11, 27.3%), one patient used ibandronate (150 mg os monthly) (1/11, 9.1%), one patient used risedronate (35 mg weekly) (1/11, 9.1%), three patients assumed minodronate (50 mg os monthly) (3/11, 27.3%) and one patient used Tyrosine kinase inhibitor (Imbrutinib, 420 mg os once daily) (1/11, 9.1%).

The mean duration of BMA therapy at presentation was 12 months for high-dose denosumab therapy, 50 ± 50 months (median 24 months) for alendronate therapy, and 60 ± 25.9 months (median 72 months) for minodronate therapy.

The mandible was the most OSCC frequently affected site (9/11, 81.8% vs. 2/11, 18.2% of lesions on the upper jaw). The bone exposure was observed in four cases (4/11, 36.4%). Mucosal dehiscence and soft tissue swelling characterized the other cases. Radiological examinations showed cortical erosion in most cases. Due to the unspecific clinical and radiological features, patients underwent incisional biopsy. No patients were found to have MRONJ. Histological diagnosis of OSCC was performed in all cases.

In the histological evaluation of the incisional biopsies performed, the diagnosis of OSCC was made based on the morphological finding of atypical epithelial neoformed tissue with the following characteristics: large nests, cords, and islands of cells with pink cytoplasm and round, often hyperchromatic nuclei; mitotic figures (especially in atypical forms); in poor differentiated OSCC cases, nuclear and cellular pleomorphism, nuclear hyperchromasia, and abundant mitotic figures were observed with, sometimes, small islands or individuals cells at the invasive front.

**Figure 1:**
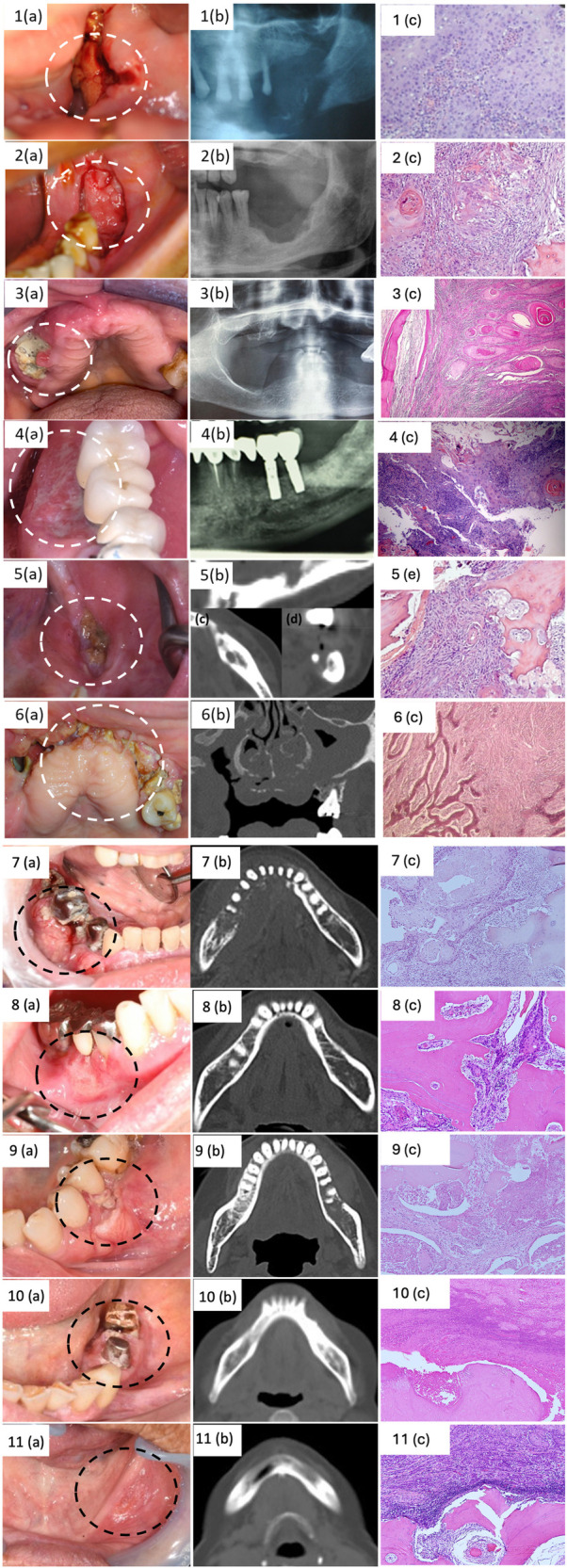
Clinical, radiological and histological features of included cases.

The main clinical, radiological and histological features were reported in Figure 1.

Below we present a detailed description of the 11 cases of OSCC mimicking MRONJ.

### 3.1 Case #1

A 90-year-old non-smoker woman with osteoporosis was treated with ibandronate for 10 years (once-monthly oral ibandronate 150 mg). Six months before the examination, she underwent dental extraction of tooth 3.6. After the tooth extraction, due to the presence of a non-healing post-extraction socket, the patient underwent bony curettage and antiseptic mouthwashes at a different institution for 3 months. Then, she was referred to the Maxillofacial Surgery Unit of Ragusa, where the extraoral examination revealed the presence of soft tissue swelling and high-volume lymph nodes in the left neck. Intraoral examination highlighted bone exposure and high granulation tissue of the posterior left portion of the mandible; additionally, she reported paresthesia in the lower left hemi lip. Orthopantomography showed extensive osteolysis, with a pathological fracture of the left mandibular body. An incisional biopsy was performed, and the histological examination revealed the OSCC diagnosis. Subsequently, the patient was referred to the Department of Oncology for management. Two years later, the patient died due to natural causes.

### 3.2 Case #2

A 72-year-old non-smoker woman with osteoporosis was treated with alendronate for more than 10 years (once-weekly oral alendronate 70 mg). Five months before the examination, she underwent dental extraction of teeth 3.6 and 3.7. After the surgery, due to the presence of a non-healing post-extraction socket, she underwent bony curettage and antiseptic mouthwashes at a different institution for 2 months. She was referred to the Maxillofacial Surgery Unit of Ragusa, where the extraoral examination revealed the presence of high-volume lymph nodes in the left neck. Intraoral examination highlighted bone exposure and soft tissue swelling of the left jaw. The patient did not refer paraesthesia of the lower lip. An incisional biopsy was performed and showed poor differentiated squamous carcinoma. Subsequently, the patient was referred to the Department of Oncology for management. The patient died a year later due to the progression of the oncological disease.

### 3.3 Case #3

A 71-year-old former smoker of 8 cigarettes per day for 40 years woman, affected by metastatic left-breast cancer treated with surgery (left breast mastectomy) followed by chemotherapy and antiresorptive therapy for the bone metastasis with high doses of Denosumab (120 mg every 4 weeks for 12 months). She was referred to the Maxillofacial Surgery Unit of the University of Campania “L. Vanvitelli”, for the 6-month routine oral and maxillofacial examination for patients in treatment with bisphosphonates. The extraoral examination highlighted regional soft tissue inflammatory swelling of the right cheek and difficulties in opening the mouth and chewing. The intraoral examination showed bone exposure associated with an intraoral fistula characterized by purulent discharge and gingival inflammation in the upper left jaw (region 1.5). CT scan of the upper and lower maxilla without contrast, showed extensive osteolysis, and necrotic bone in the region 1.4–1.7 with the complete obliteration of the maxillary sinus for sinusitis. The patient underwent surgical treatment, and a biopsy was performed. The diagnosis of OSCC was confirmed and the patient was referred to the Department of Oncology for management. Following surgical excision and radiotherapy, the patient is currently under regular follow-up with no signs of recurrence.

### 3.4 Case #4

A 75-year-old non-smoker man affected by chronic lymphocytic leukemia was treated with Imbrutinib (Tyrosine kinase inhibitor) for 14 months. The patient came to our attention at the Oral Medicine Unit at the University Hospital “Paolo Giaccone” in Palermo (Italy) due to the presence of a new lesion in the lower left jaw for about 1 month. His personal history includes hypertension, diabetes, chronic lymphocytic leukemia (diagnosed in 1989), bladder carcinoma, and benign prostatic hyperplasia. His medical history includes Xatral, Rosuvastatin, Aspirin, Glucophage, Ramipril, and Imbrutinib. The extraoral examination did not reveal any abnormalities. Physical examination revealed the presence of a lesion with irregular margins and surface, granular appearance, approximately 2 cm in diameter, involving the lingual and vestibular masticatory mucosa in correspondence with teeth 3.4 and 3.5. An incisional biopsy was performed on lingual and vestibular mucosa and the diagnosis of OSCC was performed. Therefore, the patient underwent extraction of the root remnants of teeth 4.6 and 4.7 and he was referred to the Oncology Unit and Plastic Surgery Unit for management. Currently, the patient has been in follow-up for 6 months.

### 3.5 Case #5

A 62-year-old non-smoker woman was referred to the sector of Oral Medicine Unit at the University Hospital “Paolo Giaccone” in Palermo (Italy) for swelling in his left mandible and severe pain. In 2006, the patient was diagnosed with an OSCC (grade 1) on the posterior part of the left body of the mandible in a different institution; afterward, she underwent marginal mandibular resection. Additionally, she was affected by chronic drug-induced pancreatitis and osteoporosis, and she had been treated from 2016 to 2018 with alendronate (once-weekly oral alendronate 70 mg), and steroids. The patient was partially edentulous, she reported mandibular rehabilitative treatment by 3 dental implants in 2019; all of them failed to integrate after 2 months from implant placement. The patient reported that she developed numerous episodes of swelling and pain in the implant site after the fixtures' loss. Intraoral examination revealed a large area of exposed necrotic bone of the posterior left part of the mandible. CT scan showed a diffuse osteosclerotic pattern, non-healing post-surgical site, and cortical disruption. All clinical and radiological features were compatible with a diagnosis of MRONJ; however, due to the clinical aspects of the lesions and the medical history (i.e., previous OSCC), an incisional biopsy for histological examination was promptly performed. Histological examination confirmed the diagnosis of OSCC; appropriate oncological management was therefore started.

### 3.6 Case #6

A 64-year-old non-smoker woman, affected by metastatic right-breast cancer treated with surgery (right breast mastectomy) followed by chemotherapy and antiresorptive therapy for the bone metastasis with high doses of Denosumab (120 mg every 4 weeks for 12 months). The patient was referred to the Maxillofacial Surgery Unit of the University of Campania “L. Vanvitelli”, for the 6-month routine oral and maxillofacial examination for patients in treatment with bisphosphonates. The extraoral examination highlighted regional soft tissue inflammatory swelling of the right cheek and difficulties in opening the mouth and chewing. The intraoral examination revealed the presence of bone exposure on the upper jaw region with purulent discharge and gingival erosion. CT scan showed extensive osteolysis, necrotic bone in the region 1.1–1.8 with the complete invasion and erosion of the right maxillary sinus, and the presence of bilateral lymph nodes increased in volume. Therefore, the patient underwent an incisional biopsy. The diagnosis of OSCC was obtained. Once the diagnosis was communicated, the patient preferred to go to another hospital to continue the therapeutic process. This section may be divided by subheadings. It should provide a concise and precise description of the experimental results, their interpretation, as well as the experimental conclusions that can be drawn.

### 3.7 Case #7

A 76-year-old non-smoker woman, affected by osteoporosis was treated with risedronate (17.5 mg risedronate once a week) for 5 years. The patient was referred to the Department of Oral and Maxillofacial Surgery of Dokkyo Medical University School for swelling in the right side of mandible. The patient presented with erythema and diffuse swelling on the molar region of the right mandible. The intraoral examination revealed the presence of a granular and easily hemorrhagic tumor growth in the molar region of the right mandible (70 mm × 37 mm). There was no evidence of paresthesia of the right-mental region and lower lip. An incisional biopsy was performed and showed oral squamous carcinoma. Sectional mandibular resection and the reconstruction by titanium plate was performed, followed by cervical lymph nodes dissection on the right side. The patient continues to have a good postoperative course and there are no findings for MRONJ.

### 3.8 Case #8

A 64-year-old non-smoker woman affected by osteoporosis was treated with minodronate (50 mg once a week) for 2 years. The patients underwent periodic follow-up visits at Department of Oral and Maxillofacial Surgery of Dokkyo Medical University School since 2014 for the lichen planus history. At the last follow-up visit, the patients presented with erosions and spontaneous bleeding of the right mandibular mucosa. A biopsy was taken and a diagnosis of moderately differentiated squamous cell carcinoma on the mandibular gingival was obtained. She underwent marginal mandibular resection and did well, but died of other illnesses.

### 3.9 Case #9

A 70-year-old non-smoker woman affected by osteoporosis was treated with minodronate (50 mg once a week) for 6 years. She was referred to Department of Oral and Maxillofacial Surgery of Dokkyo Medical University School for swelling and pain in her left mandibular gingiva. She underwent oral biopsy and diagnosis of oral squamous cell carcinoma was performed. Sectional mandibular resection and the reconstruction by titanium plate was performed, and chemotherapy was done postoperatively. The patient continues to have a good postoperative course and there are no findings for MRONJ.

### 3.10 Case #10

A 78-year-old non-smoker woman affected by osteoporosis was treated with minodronate (50 mg once a week) for 7 years. The patients underwent periodic follow-up visits at Department of Oral and Maxillofacial Surgery of Dokkyo Medical University School since 2019 for the history of mucosal dysplasia of the left-sided mandibular gingiva. At the last follow-up visit, the intraoral examination revealed the presence of a mass-like lesion appeared on the perigingival mucosa between 3.5 and 3.6. A biopsy was performed and a diagnosis of OSCC was made. She underwent marginal mandibular resection and did well. The patient continues to have a good postoperative course and there are no findings for MRONJ.

### 3.11 Case #11

An 81-year-old nonsmoker woman was referred to Department of Oral and Maxillofacial Surgery of Dokkyo Medical University School for the pain in her left mandibular gingiva for about 2 months and suspected a denture-induced ulcer. The patients had been taking alendronate (35 mg once a week) for the last 6 months due to osteoporosis. An incisional oral biopsy was performed and the diagnosis of OSCC was carried out. The patient underwent a cervical lymph nodes dissection in addition to sectional mandibular resection and the reconstruction by titanium plate of the left side mandible. The patient continues to have a good postoperative course and there are no findings for MRONJ.

## 4 Discussion

Despite recent advances in diagnosis and treatment, the incidence of OSCC is increasing and the mortality rate is still high (4, 9). Therefore, early diagnosis and treatment are still crucial to improve prognosis. Notably, when an accurate diagnosis occurs at the early stage, the 5-year survival rate is higher than 90%, underscoring the pivotal role of timely diagnosis and treatment in improving patient outcomes (18). Primary care physicians and dental practitioners are integral contributors to the primary and secondary prevention of OSCC. In addition to the importance of controlling and eliminating known risk factors (e.g., smoking cessation, reduction of alcohol consumption), oral health specialists should be vigilant in identifying the initial clinical indicators during dental visits to contribute to the early-stage diagnosis of OSCC. Moreover, it is fundamental to consider oral cancer in the differentiation of clinical manifestations of other oral disorders (16).

The great paradox of OSCC is that, despite easy access to the oral region for medical examination and improvements in therapeutic approaches to the disease, its mortality rate remains high (ranging between 55% and 50%) and, even more unexpectedly, like cancers that occur in less accessible areas, such as the colon, cervix, and breast (19, 20).

OSCC occurs with early visible and symptomatic mucosal changes, including ulcers, and oral potentially malignant disorders (e.g., erythroplakia, leukoplakia, oral lichen planus), accompanied by bleeding and pain (21, 22).

A thorough clinical exam for suspected oral cancer involves a quick, non-invasive visual inspection, taking only 5 min during routine medical/dental checks (23).

Despite the simplicity of this test, a significant number of patients are diagnosed at an advanced stage (24). This diagnostic delay is also associated with a misinterpretation of the initial signs, which on some occasions are associated with minor oral problems such as trauma, infectious processes, or conditions related to the prosthesis or other dental factors (25–27).

In the present study, all cases suspected of MRONJ were oral squamous cell carcinoma (OSCC) and they exhibited mucosal alterations, such as inflammation, swelling, dehiscence, and purulent discharge. Bone exposure was observed in four cases (36.4% of the total). It has been demonstrated that a percentage of patients affected by MRONJ go undiagnosed as they have no bone exposure (28). For this reason, it is important to highlight that the absence of exposed bone in the oral cavity should not make us discard the chance of MRONJ (28).

Due to the generic clinical characteristics and the drug history of the patients, at first, the diagnostic suspicion was for all MRONJ.

Indeed, one of the severe diseases that can manifest with not specific signs and symptoms referable also to OSCC is MRONJ.

MRONJ is a drug adverse reaction that can greatly affect the quality of life of patients if not promptly diagnosed and treated (14).

The main MRONJ risk populations are principally cancer patients with bone metastases, patients affected by multiple myeloma or patients affected by Giant Cell Tumour of Bone, commonly receiving high-dose (HD) of BMAs; secondly, breast cancer or prostate cancer patients suffering from osteoporosis generally without bone metastases receiving low-dose (LD) of BMAs for cancer-treatment-induced bone loss (CTIBL) and patients suffering from osteoporosis and other non-malignant diseases receiving LD-BMA therapy (14).

In the present series, the patients studied were predominantly osteoporotic patients, followed by patients affected by breast cancer with bone metastases and a patient affected by chronic lymphocytic leukemia.

According to the SIPMO-SICMF definition, patients may be considered to have MRONJ if all the following characteristics are present (13):

• Current or previous treatment with BMAs and/or antiangiogenic agents (AAs).
• Clinical and radiological findings of progressive bone destruction.
• No history of radiation therapy to the jaws or the presence of primary oral malignancy or metastatic disease to the jaws.

Sometimes the early diagnosis of MRONJ could be a difficult task as different disorders present with similar clinical manifestations, including osteomyelitis, primary malignant tumors of the jawbone, and metastatic disease. Exclusion of these conditions and description of MRONJ lesions require imaging (29), but, unfortunately, the clinical and radiological signs of MRONJ are often nonspecific.

Patients affected by MRONJ may present clinical symptoms common to other diseases, especially OSCC. The main clinical and radiological features shared and unshared between MRONJ and OSCC are reported in Tables 2.1, 2.2 respectively.

**Table 2.1:** Clinical features shared and unshared between OSCC and MRONJ.

Disease	Shared clinical features	Unshared clinical features
OSCC	• Bone exposure • Halitosis • Hypoesthesia/paraesthesia of the lips • Pain • Non-healing post-extraction socket • Soft tissue swelling • Sudden dental/implant mobility • Loose tooth/implant • Toothache • Mucosal inflammation • Intraoral fistula	• Ulceration with or without raised exophytic margins • Nodularity • Fixation to underlying tissues • Lymph node enlargement
MRONJ		• Abscess • Cutaneous fistula • Fluid discharge from the nose • Intraoral fistula • Mandible fracture (fragment mobility) • Mandibular deformation • Spontaneous loss of bone fragments

**Table 2.2:** Radiological features shared and unshared between OSCC and MRONJ.

Disease	Shared radiological features	Unshared radiological features
OSCC	• Cortical erosion • Osteolytic changes • Osteolysis extending to the maxillary sinus • Osteosclerosis of adjacent bones • Persistent post-extraction socket	• Resorption of tooth roots • The teeth appear floating
MRONJ		• Diffuse bone marrow sclerosis • Focal bone marrow sclerosis • Opacified maxillary sinus • Pathologic fracture • Periodontal space widening • Periosteal reaction • Sequester formation • Sinus tract • Thickening of the alveolar ridge • Thickening of the lamina dura • Thickening of the inferior alveolar nerve canal • Trabecular thickening

It is important to underline that MRONJ and OSCC are predominantly assessable through distinct radiological imaging modalities.

Concerning MRONJ, there is currently no full consensus on the preferred imaging technique for diagnosis and screening (30–33).

Plain radiographs, providing an immediate view of the lesions, offer a basic understanding of the pathological condition and reveal bone changes suggestive of MRONJ, especially appreciable in the advanced stage (13).

Second-line CT-based imaging modalities, including cone-beam computed tomography (CBCT) and multidetector CT (MDCT), are much more accurate than plain radiographs, providing more precise and especially 3D images (31).

These methods are essential for uncovering early signs of MRONJ, facilitating early diagnosis, and accurately defining the stage and treatment options (13).

In selected cases, Magnetic Resonance Imaging (MRI), bone scintigraphy, Positron Emission Tomography (PET), and Single Photon Emission Computed Tomography can be useful tools for patient evaluation, but only when already in possession of the patient's (34, 35).

The diagnostic workflow of patients suspected of OSCC consists of radiological studies and incisional biopsy. MRI with contrast and/or CT with contrast are indicated to determine the anatomic extent of the disease (35). Moreover, a dental evaluation through a panoramic dental x-ray should be performed to evaluate oral health in patients expected to receive postoperative radiotherapy of the head and neck district. Other radiological investigations, including PET and lymph node ultrasound, can be useful in evaluating lymph node involvement and the presence of any metastases (33).

Although some radiological investigations are potentially useful for the differential diagnosis of both diseases, the most suitable exams are usually CBCT for MRONJ and MRI for OSCC.

To our knowledge, few studies have described malignant oral lesions in patients assuming MRONJ-related medication, including primary OSCC, metastases, or multiple myeloma.

Some studies reported cases of OSCC with clinical and radiological features resembling MRONJ in patients undergoing LD-BMA therapy for osteoporosis (36–38).

The most common clinical features were bone exposure and hyperplastic epithelium, and the radiological feature was a mandibular osteolytic lesion.

Gander et al. reported three cases of patients receiving high-dose anti-resorptive therapy for metastatic malignancies presenting with suspicious osteonecrosis of the jaw; histologic analysis revealed malignant disease in all cases (39).

Beattie et al. described a case of a 55-year-old woman with a medical history of methotrexate, adalimumab, and alendronic acid, where the clinical appearance of OSCC in the mandible mimicked a non-healed socket, common in patients affected by MRONJ (40).

Arduino et al. reported on an OSCC that appeared adjacent to an area diagnosed as MRONJ (41).

Cases of jaw metastases in patients taking ONJ-related drugs have also been described (42, 43).

Bedogni et al. reported cases of early jaw metastases of breast cancer and diffusely metastatic thyroid medullary carcinoma in patients undergoing zoledronate and pamidronate therapy, respectively (44). In the study performed by Bedogni et al., clinical and radiological signs of jaw metastases were missing, and the initial suspected diagnosis was bisphosphonates-associated osteonecrosis of the jaw. Patients underwent surgical resection, bone biopsy, and subsequent definitive diagnosis due to failure of the conservative procedure treatment for bisphosphonates-associated osteonecrosis of the jaw (44).

Carlson et al. described cases of patients developing both Bisphosphonate-Related Osteonecrosis of the Jaw (BRONJ) and metastatic cancers in the jawbones, including multiple myeloma, metastatic breast cancer, undifferentiated carcinoma, carcinoid, and renal cell carcinoma (45).

Corsi et al. reported synchronous osteonecrosis and breast cancer metastasis in a patient treated with zoledronic acid for bone metastasis (46).

Favia et al. reported metastatic breast cancer in MRONJ associated with dental implants (47), and Frei et al. observed distant metastasis from prostate adenocarcinoma in a patient receiving zoledronate for prostate cancer and bone metastases (48).

Junquera et al. reported multiple myeloma infiltration in the maxilla in patients undergoing zoledronate therapy (49).

Multiple myeloma, breast, and prostate cancer are tumors that frequently metastasize to bone and require bisphosphonate treatment. MRONJ is a well-known potential complication of bisphosphonate treatment. Thus, in cancer patients treated with BMAs, the concurrence of malignancy disease and MRONJ should not be surprising (45).

Moreover, to the best of our knowledge, in literature also a few cases of concurrent OSCC and MRONJ have been reported, including in patients under LD-BMA therapy for osteoporosis (50). This underscores the complexity and rarity of such conditions and the critical importance of histologic evaluation in differentiating these pathologies.

Furthermore, in cancer patients, oral lesions are more likely to prompt the clinician to consider the possibility of a secondary tumor, a scenario that does not occur in patients with osteometabolic pathology taking BMA medications.

Despite this, the present study highlights, based on reported cases and literature data, the need to consider the possibility of primary cancer, especially in patients undergoing LD-BMA therapy for osteoporosis, as these individuals have a lower probability of developing MRONJ compared to cancer patients (13).

For this latter reason and given the nonspecific clinical and radiological characteristics of both MRONJ and oral malignant lesions, we recommend, also in patients at risk of MRONJ, bone and/or oral mucosa biopsy in cases of suspected primary oral malignancy or metastatic bone disease.

## 5 Conclusion

Although bone biopsy is commonly considered unnecessary for the diagnosis of MRONJ, our findings highlight the importance of selectively performing this procedure in patients undergoing BMA therapy. This is recommended not only in patients undergoing HD-BMA therapy for cancer disease but also in patients undergoing LD-BMA therapy for osteoporosis, although they have a relatively lower risk of developing MRONJ. Moreover, imaging is fundamental in revealing the lesion extension, its characteristics, and bone invasion, assisting the diagnosis process, staging, and subsequent treatment. Our study emphasizes the need for a targeted and personalized approach in diagnostic protocols to minimize the risk of overlooking malignancy and reduce complications.

## Data Availability

The raw data supporting the conclusions of this article will be made available by the authors, without undue reservation.
